# Modified *Spirulina maxima* Pectin Nanoparticles Improve the Developmental Competence of In Vitro Matured Porcine Oocytes

**DOI:** 10.3390/ani11092483

**Published:** 2021-08-24

**Authors:** Pantu-Kumar Roy, Ahmad-Yar Qamar, Bereket-Molla Tanga, Seonggyu Bang, Gyeonghwan Seong, Xun Fang, Ghangyong Kim, Shan-Lakmal Edirisinghe, Mahanama De Zoysa, Do-Hyung Kang, Islam M. Saadeldin, Jongki Cho

**Affiliations:** 1 College of Veterinary Medicine, Chungnam National University, Daejeon 34134, Korea; vetpantu88@gmail.com (P.-K.R.); ahmad.qamar@uvas.edu.pk (A.-Y.Q.); tanga@o.cnu.ac.kr (B.-M.T.); bangsk97@o.cnu.ac.kr (S.B.); 202050377@o.cnu.ac.kr (G.S.); fx2442@o.cnu.ac.kr (X.F.); gykim1007@gmail.com (G.K.); shan.lakmal09011@gmail.com (S.-L.E.); mahanama@cnu.ac.kr (M.D.Z.); islamms@zu.edu.eg (I.M.S.); 2 Department of Clinical Sciences, College of Veterinary and Animal Sciences, Jhang 35200, Pakistan; 3 Faculty of Veterinary Medicine, Hawassa University, Hawassa 05, Ethiopia; 4 Jeju Marine Research Center, Korea Institute of Ocean Science and Technology (KIOST), Jeju 63349, Korea; dohkang@kiost.ac.kr; 5 Department of Ocean Science, University of Science and Technology (UST), Jeju 63349, Korea

**Keywords:** *Spirulina maxima* pectin, nanoparticles, porcine, embryos, development

## Abstract

**Simple Summary:**

Poor in vitro embryo development is a major obstacle in porcine assisted reproduction. In the current study, we utilized modified *Spirulina maxima* pectin nanoparticles as a supplement to improve porcine in vitro maturation medium. Results showed that modified *Spirulina maxima* pectin nanoparticles at 2.5 µg/mL improved oocyte maturation in form of first polar body extrusion, reduced oxidative stress, and increased the developmental competence of the oocytes after parthenogenetic activation and somatic cell nuclear transfer. Moreover, the relative transcripts quantification showed significant increase in the pluripotency-associated transcripts in the resultant cloned embryos after modified *Spirulina maxima* pectin nanoparticles supplementation. Therefore, we provide an optimum in vitro maturation condition to improve the in vitro embryo production in porcine.

**Abstract:**

Molecular approaches have been used to determine metabolic substrates involved in the early embryonic processes to provide adequate culture conditions. To investigate the effect of modified *Spirulina maxima* pectin nanoparticles (MSmPNPs) on oocyte developmental competence, cumulus–oocyte complexes (COCs) retrieved from pig slaughterhouse ovaries were subjected to various concentrations of MSmPNPs (0, 2.5, 5.0, and 10 µg/mL) during in vitro maturation (IVM). In comparison to the control, MSmPNPs-5.0, and MSmPNPs-10 groups, oocytes treated with 2.5 µg/mL MSmPNPs had significantly increased glutathione (GSH) levels and lower levels of reactive oxygen species (ROS). Following parthenogenetic activation, the MSmPNPs-2.5 group had a considerably higher maturation and cleavage rates, blastocyst development, total cell number, and ratio of inner cell mass/trophectoderm (ICM:TE) cells, when compared with those in the control and all other treated groups. Furthermore, similar findings were reported for the developmental competence of somatic cell nuclear transfer (SCNT)-derived embryos. Additionally, the relative quantification of POU5F1, DPPA2, and NDP52 mRNA transcript levels were significantly higher in the MSmPNPs-2.5 group than in the control and other treated groups. Taken together, the current findings suggest that MSmPNP treatment alleviates oxidative stress and enhances the developmental competence of porcine in vitro matured oocytes after parthenogenetic activation and SCNT.

## 1. Introduction

Nanotechnology is a promising technique owing to its increasing applicability in economically areas, such as agriculture, industry, medicine, and public health [1,2]. Nanotechnology has been applied to improve animal production and health using different approaches [3,4]. Nanoparticles (NPs) have been used in disease diagnosis, drug administration, animal nutrition, reproduction, and food safety [5,6]. Because of their unique and distinctive physicochemical properties that differ significantly from bulk materials of the same composition, nanomaterials are being created for use in a wide range of commercial goods worldwide. There are some physicochemical properties give synthetic NPs features and a higher surface reactivity than their counterparts of the regular bulk materials; such as the minute size (increased surface area and size distribution), purity, surface reactivity, solubility, shape, and aggregation [7,8].

NPs, such as lipid core NPs, supplemented during in vitro maturation (IVM), have previously been shown to improve oocyte quality, embryo cleavage, and blastocyst rates [9]. Furthermore, chitosan NPs efficiently prevent oxidative damage to oocytes [10]. In a previous study, we examined the effects of chitosan NP supplementation during IVM on porcine oocyte developmental competence and pre-implantation development in parthenogenetic and cloned embryos [11]. NPs containing various antioxidant materials can scavenge reactive oxygen species (ROS) and thus protect cellular molecules, such as lipids, proteins, and DNA, from oxidative stress, [11,12,13]. Therefore, many NPs are currently being used to improve the entire process of in vitro embryo production (IVP), such as gametes cryopreservation, oocyte in vitro maturation, and embryo culture [12,14,15,16,17], but some of these NPs exert toxic effects [18,19,20,21,22,23,24,25]. To overcome these toxic effects, plant-derived nanoparticles are biologically safe and applicable for improving the quality of oocytes and subsequent embryo development [1,26].

Despite the chemical activation of in vitro fertilization (IVF), the therapeutic properties of plant extracts or their secondary derivatives on the regulation of folliculogenesis have been extensively studied under both in vitro and in vivo conditions [27]. In particular, plant polysaccharides have shown developmental competence of in vitro matured mouse oocytes by protective effects, such as reducing endoplasmic reticulum (ER) stress, preventing cell death, and activating both phosphatidylinositol 3-kinase (PI3K)/ Akt (protein kinase B, AKT) and mitogen-activated protein kinase (MAPK3/1) signaling [28].

Pectin is a cell wall ingredient in terrestrial plants, and feeds incorporated polysaccharides from livestock animals. The three major pectic polysaccharides are homogalacturonan (HG), rhamnogalacturonan-I (RG-I), and rhamnogalacturonan-II (RG-II) [29]. In clinical studies, pectin has been shown to have a broad range of immunomodulatory activities due to different molecular characteristics, such as source, extraction technique, degree of esterification (DE), degree of acetylation, and chemical modifications [30,31]. Among the other farm animals, pectin has shown a beneficial impact on pig nutrition and health contexts, which was performed in vivo, ex vivo, and in vitro studies [32]. The development of reproductive performance using crude extracts of microalgae has been widely discussed in livestock animals [33,34,35,36]. Microalgae *Spirulina* is a photosynthetic cyanobacterium that has been used to improve reproductive efficiency in in vivo bovine studies [33]. Recently, *S. maxima*-based pectin (SmP), and their modified two products namely, modified SmP (MSmP) and its nanoparticles (MSmPNPs) were investigated for different bioactivities in in vitro and in vivo studies [37,38,39]. Modification of SmP remarkably enhanced their functionality via physicochemical properties.

The production of cloned and transgenic pigs is a crucial step in xenotransplantation [40]. For successful production of these pigs, oxidative stress of recipient oocytes must be decreased during in vitro maturation (IVM), which can reprogram the gene-modified donor cells appropriately [41,42,43]. The goal of this study was to determine how MSmPNPs supplementation during IVM affected porcine oocyte developmental competence and pre-implantation development in parthenogenetic and cloned embryos. Consequently, this study was conducted to investigate the effects of MSmPNP supplementation on porcine oocyte maturation to reveal novel functional properties that enhance porcine IVP through parthenogenesis and SCNT. Intracellular glutathione (GSH) and ROS levels in the oocytes, pre-implantation embryo development, and the expression of a reprogramming-related gene were investigated.

## 2. Materials and Methods

### 2.1. Chemicals and Reagents

Unless otherwise stated, all chemicals and reagents were acquired from Sigma-Aldrich (St. Louis, MO, USA).

### 2.2. Preparation of MSmPNPs

*S. maxima* based MSmP was provided by the Jeju Marine Research Institute, Korea Institute of Ocean Science and Technology (KIOST), Jeju Special Self-Governing Province, Republic of Korea. The particle size of MSmP was further reduced by mechanical sonication. Briefly, MSmP was dissolved in deionized water and sonicated under an amplitude of 30%, 10:10 s pulse at 4 °C for 30 min (Sonics & Materials. Inc. Newtown, CT, USA). Sonicated MSmP was centrifuged at 3500 rpm for 15 min to collect MSmPNPs in the supernatant [39]. The average particle sizes of MSmp and MSmPNPs are 542.4 nm and 78.6 nm, respectively. Zeta potential of MSmp and MSmPNPs are −22.8 mV and −19.8 mV, respectively.

### 2.3. Oocyte In Vitro Maturation and MSmPNPs Treatment

The procedure for collecting porcine oocytes and IVM was performed according to our previous investigations [44]. Briefly, apparently healthy porcine ovaries were obtained within 4 h of slaughter at a local abattoir. Follicular fluid from 3–8 mm in diameter was aspirated into a 15 mL conical tube (Corning, Acton, MA, USA) using a 10 mL syringe and an 18-gauge needle. The fluid was rinsed with HEPES-buffered Tyrode’s (TLH) medium (119 mM NaCl, 5 mM KCl, 25 mM HEPES buffer, 2 mM CaCl2, 2 mM MgCl2, 6 g/liter glucose, adjust pH to 7.4 with NaOH) containing 0.05% (*w*/*v*) polyvinyl alcohol (PVA) after the oocytes settled for 5 min. A stereomicroscope was used to select cumulus–oocyte complexes (COCs) with at least three layers of compact cumulus cells and homogenous ooplasm. COCs were washed three times with TLH–PVA and then with Dulbecco’s phosphate-buffered saline (DPBS; Gibco, Life Technologies, Grand Island, NY, USA) modified with 0.4% bovine serum albumin (BSA) (mDPBS). The IVM medium consisted of TCM-199 (Gibco) supplemented with 10% (*v*/*v*) of porcine follicular fluid (pFF), 0.6mM cysteine, 0.91mM sodium pyruvate, 75 µg/mL kanamycin, 10 ng/mL epidermal growth factor (EGF), 1 µg/mL insulin, 10 IU/mL human chorionic gonadotrophin (hCG; Intervet International BV, Boxmeer, Holland), and 10 IU/mL pregnant mare serum gonadotrophin (PMSG). COCs were then incubated in IVM medium (50 COCs/500 µL) at 39 °C in a humidified environment containing 5% CO_2_. COCs were moved into hormone-free IVM medium after 22 h of incubation and cultured in four-well multi dishes (SPL, Pocheon, South Korea) for another 22 h under the same conditions. The oocytes in the control group were not supplemented to MSmPNPs during IVM, while the remaining oocytes were separated into three groups and treated with varied doses of MSmPNPs (2.5, 5.0, and 10 µg/mL) for the first 22 h. Gentle pipetting of COCs in 0.5 mg/mL hyaluronidase solution (Catalog no. H7630, in PBS) in PBS was used to denude the in vitro matured oocytes. The oocyte morphology and appearance of the polar body in the perivitelline space were used to evaluate oocyte maturation, where oocytes in the metaphase II (MII) stage showed the first polar bodies with a dark distinct cytoplasm.

### 2.4. Measurement of Intracellular GSH and ROS Levels

**Figure 1:**
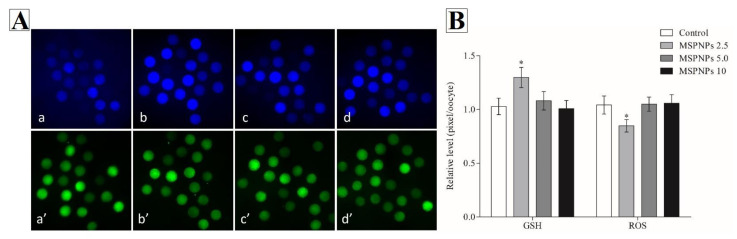
Evaluation of glutathione and reactive oxygen species of in vitro matured porcine oocytes at different concentrations of MSPNPs. (**A**) Oocytes were stained with (**a**–**d**) Cell Tracker Blue and (**a’**–**d’**) 2′,7′-dichlorodihydrofluorescein diacetate (H2DCFDA) to detect intracellular levels of glutathione (GSH) and reactive oxygen species (ROS), respectively, whereas (**a**,**a’**) control and (**b**,**b’**) MSPNPs-2.5, (**c**,**c’**) MSPNPs-5.0, and (**d**,**d’**) MSPNPs-10 matured oocytes. (**B**) Effects on intracellular levels of GSH and ROS in vitro matured porcine oocytes. * Indicates that there is a significant difference in the GSH and ROS levels between each group (*p* < 0.05). GSH samples, n = 60; ROS samples, n = 60. The experiment was independently replicated three times.

The levels of intracellular GSH and ROS in in vitro-matured oocytes were measured as previously described [11,45]. The intensity of green fluorescence of 2,7-dichlorodihydrofluorescein diacetate was used to estimate intracellular ROS levels (H_2_DCFDA; Invitrogen Corporation, Carlsbad, CA, USA). After 44 h of IVM, intracellular GSH levels were measured using CellTracker^TM^ Blue, which contains the blue fluorescent dye 4-chloromethyl-6,8-difluoro-7-hydroxycoumarin (CMF_2_HC, Invitrogen Corporation). Twenty oocytes from each group were incubated in TLH-PVA supplemented with 10 µM H_2_DCFDA and 10 µM Cell Tracker Blue in the dark for 30 min. Finally, oocytes were analyzed using an epifluorescence microscope with UV filters (×200 magnification; Leica Application Suite X; Leica Microsystems, Wetzlar, Germany) (460 nm for ROS and 370 nm for GSH) (Figure 1a). The intensity of fluorescence was measured, and the photos were stored as TIFF graphic files for subsequent examination. The fluorescence intensity of the oocytes was standardized to that of the control oocytes using ImageJ software (version 1.41; National Institutes of Health, Bethesda, MD, USA).

### 2.5. In Vitro Embryo Production

Oocytes were parthenogenetically activated (PA) or used as karyoplasts for SCNT, with some modifications to our previous studies [45]. For PA, COCs were cultured in IVM medium 22 h and hormone-free IVM medium for 22 h and were then exposed to 0.1% (*w*/*v*) hyaluronidase. Cumulus cells were then and repeatedly pipetted gently. The mature, good-quality oocytes were parthenogenetically triggered with two 60 µsec direct current (DC) pulses of 120 V/cm in 280 mM mannitol solution with 0.01 mM CaCl_2_ and 0.05 mM MgCl_2_ using a BTX 2001 Electro-cell Manipulator (BTX Harvard Apparatus, San Diego, CA, USA). For SCNT, a primary culture of donor cells was made from the kidney cells of an aborted cloned male pig at 50 days of gestation, which was chopped into small pieces and centrifuged three times. The culture medium comprised Dulbecco’s Modified Eagle Medium (Gibco) with 10% (*v*/*v*) fetal bovine serum in a 60 mm tissue culture plate until a monolayer of cells was established with 70–80% confluency. For 48–72 h, donor cells in the G0/G1 stage of the cell cycle were synchronized by serum starvation. Donor cells were prepared by resuspending trypsinized cultured cells prior to nuclear transfer with 0.4% (*w*/*v*) BSA (TLH). Mature COCs were denuded as mentioned above. The denuded oocytes were then incubated in 5 µg/mL Hoechst 33342 medium for 15 min. A 17 µm beveled glass pipette was used to enucleate and extract polar bodies from metaphase II oocytes. A single cell was injected into the perivitelline space. In a 280 mM mannitol solution with a low Ca^+2^ concentration (0.001 mM), reconstructed SCNT oocytes were electrofused with two pulses of DC at 160 V/cm for 40 µs, followed by an AC of 2 V/cm for 2 s, using a BTX 2001 Electro-cell Manipulator (Harvard Apparatus, San Diego, CA, USA). Presumptive zygotes were activated in a 280 mM mannitol solution containing 0.01 mM CaCl_2_ and 0.05 mM MgCl_2_, after 30 min of fusing with two pulses of DC at 120 V/cm for 60 µs. Both PA and SCNT embryos were post-activated for 4 h with 10 µg/mL cytochalasin B and 6-dimethylaminopurine after electrical activation [46]. Activated oocytes were then washed three times in an in vitro culture medium (porcine zygote medium-5; IFP, Higashine, Yamagata, Japan) and 20 oocytes cultured in 25 µL droplets, covered with pre-warmed mineral oil, and the embryos were then cultured at 39 °C in a humidified atmosphere (5% O_2_, 5% CO_2_, and 90% N_2_). Cleavage and blastocyst formation were measured on days two and six for embryo development and blastocyst formation, respectively. Cell numbers were counted on day six to determine the total cell number, the inner cell mass (ICM), and trophectoderm (TE) expression in accordance with the differential staining methodology described in our previous studies [45].

### 2.6. Analysis of mRNA Transcript Expression by Relative Quantitative Real-Time Polymerase Chain Reaction (qRT-PCR)

A relative quantitative polymerase chain reaction (qRT-PCR) was used to analyze the mRNA expression of genes involved in nuclear reprogramming and pluripotency (*POU5F1*, *NDP52*, and *DPPA2*). The primer sequences are listed in Table 1. TRIzol reagent (Invitrogen Corporation) was used to extract total RNA from six-day-old blastocysts from the untreated (control) and treated groups [47]. Reverse transcription 2X RT Pre-Mix (BioFACT Co., Ltd., Daejeon, Korea) and oligo dT primers were used to generate complementary DNA (cDNA) from 300 ng of total RNA (Neoprobe). The absorbance of a diluted RNA samples was measured at 260 and 280 nm by NanoDrop Spectrophotometer (Thermo Fisher Scientific, Pittsburgh, PA, USA). Each RNA sample consisted of 0.5µg/µL. The following reaction settings were used for RT-qPCR: denaturation at 95 °C for 15 min and 20 s, followed by 40 cycles of annealing and extension at 60 °C for 40 s (BIOFACT Co., Ltd., Daejeon, Korea). The expression of each target gene was measured in comparison to that of the internal control gene (β-actin). The threshold cycle (Ct) at a constant fluorescence intensity was used for relative quantification of gene expression using the 2^−(ΔCt sample − ΔCt control)^ method [48]. Each value was normalized to that of β-actin to determine the normalized arbitrary values for each gene.

### 2.7. Experimental Design

The effects of supplementing porcine oocytes with or without 0, 2.5, 5.0, and 10 µg/mL MSmPNPs during IVM were examined in Experiment 1. Intracellular GSH and ROS levels were measured after 44 h of IVM. The effects of MSmPNP treatment during IVM of oocytes were investigated on the developmental competence of parthenogenetic and cloned embryos in Experiment 2. The effect of MSmPNP supplementation on the number of parthenogenetic and cloned blastocysts was investigated in Experiment 3. The effects of MSmPNP supplementation during IVM reprogramming-related genes (POU5F1, DPPA2, and NDP52) and the control gene (β-actin) in the cloned blastocysts acquired in Experiment 2 were investigated in Experiment 4.

### 2.8. Statistical Analysis

Origin software (version 8.1; OriginLab Corporation, Northampton, MA, USA) was used to analyze the data. All data are reported as mean ± standard error of the mean (SEM), with a probability (*p*) value of <0.05, regarded as statistically significant. The generalized linear model technique and one-way analysis of variance (ANOVA) were used to assess data on oocyte maturation, blastocyst development rates in PA and cloned embryos, cell number, GSH, ROS, and gene expression. Duncan’s multiple range test was used to establish significance.

## 3. Results

### 3.1. GSH and ROS Intracellular Levels Treated with/without MSmPNPs

Following oocyte maturation, the levels of intracellular GSH and ROS were measured. Mature oocytes in the MSmPNPs-2.5 group had significantly higher intracellular GSH levels (*p* < 0.05)) compared to the control, MSmPNPs-5.0 and MSmPNPs-10 groups, (Figure 1b). Furthermore, intracellular ROS levels in the MSmPNPs-2.5 group were considerably lower (*p* < 0.05)) than those in the control and other MSmPNP-treated groups.

### 3.2. The Effect of MSmPNPs on the Developmental Competence of PA Embryos

To improve the maturation, cleavage, and blastocyst development rate, varying amounts of MSmPNPs were added to in vitro maturation media. The results showed that the MSmPNPs-2.5 group significantly increased (*p* < 0.05) maturation (91.0 ± 1.0% vs. 86.5 ± 1.3% vs. 80.0 ± 0.8% vs. 83.5 ± 1.8%, respectively) compared to MSmPNPs-5.0, MSmNPS-10, and control groups (Table 2). The MSmPNPS-2.5 group displayed a significantly increased cleavage (90.5 ± 0.8% vs. 86.4 ± 1.3% vs. 80.3 ± 1.1% vs. 85.1 ± 1.3%, respectively) and blastocysts rate (34.5 ± 1.4% vs. 29.6 ± 1.7% vs. 24.9 ± 1.1% vs. 29.2 ± 1.0%, respectively) than other groups.

### 3.3. MSmPNPs Effects on Cell Number of PA Embryos

Table 3 shows a comparison of the total number of cells, ICM, TE, and ICM:TE ratio in PA embryos between the MSmPNP-treated groups and the control group. Compared to the control group (42.8 ± 1.2%), the MSmPNPs-2.5 group (48.7 ± 1.4%) showed a significant (*p* < 0.05) increase in the total cell number than the MSmPNP_S_-5.0 (44.0 ± 1.1%) and MSmPNPS-10 (40.2 ± 1.0%) groups. These results revealed that higher concentrations of MSmPNPs reduced the total cell number, the TE, ICM, and ICM:TE, while MSmPNPs-2.5 groups showed a significantly increased in these parameters.

### 3.4. MSmPNPs Effects on the Developmental Competence of Cloned Embryos

Cloned embryos produced from MSmPNPs-2.5-treated oocytes had higher development rates than the other groups (*p* < 0.05) (Table 4). Oocytes in the MSmPNPs-2.5 group had significantly higher in maturation (89.5 ± 1.8%), cleavage (88.5 ± 1.6%), and blastocysts rates (31.1 ± 1.1%) than those in the control (83.0 ± 1.5, 84.7 ± 1.0, and 24.8 ± 0.9%, respectively), MSmPNPs-5.0 (86.5 ± 1.3, 87.4 ± 2.1, and 25.9 ± 0.9%, respectively), and MSmPNPs-10 (80.5 ± 1.4, 82.6 ± 1.3, and 22.7 ± 1.4%, respectively) treated groups.

### 3.5. MSmPNPs Effects on Cloned Cell Number

Table 5 compares the total cell number, ICM, TE expression, and ICM:TE ratio of cloned embryos. In the MSmPNPs-2.5-treated oocytes group, the overall cell number (48.9 ± 1.5%), TE (74.0 ± 0.9%), ICM (26.0 ± 0.9%), and ICM:TE (35.7 ± 1.8%) were significantly higher (*p* < 0.05) than those in the control and other treated groups.

### 3.6. MSmPNPs Effects on Reprogramming-Related Gene Expression

**Figure 2:**
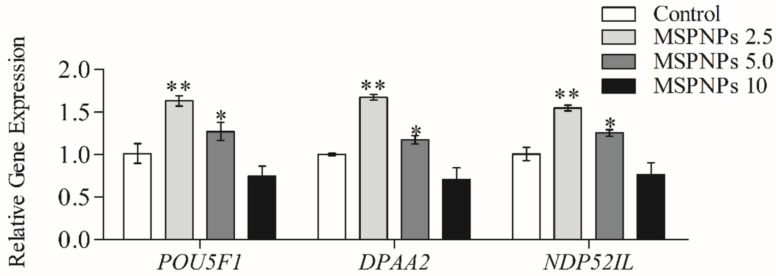
Relative expression of *POU5F1*, *DPPA2*, and *NDP52* mRNA transcripts in cloned blastocysts obtained from oocytes supplemented with varying concentrations of MSmPNPs (0, 2.5, 5.0, and 10 µg/mL) in maturation media. Each sample consisted of five blastocysts and three times replicate. * Indicates that there is a significant difference in mRNA values between each group (*p* < 0.05). ** Indicates that there is a significant difference in mRNA values at *p* < 0.01.

qRT-PCR analysis of cloned blastocyst mRNA transcripts supported the benefits of MSmPNPs-2.5 and MSmPNPs-5.0 supplementation during porcine oocyte IVM on the development of resultant embryos. The expression levels of the reprogramming-related genes, *POU5F1*, *DPPA2*, and *NDP52*, were significantly higher in cloned blastocysts produced from MSmPNPs-2.5-and MSmPNPs-5.0-treated oocytes than in MSmPNPs-10 and untreated control blastocysts. Moreover, the relative expression levels of these genes in the MSmPNPs-2.5 group were significantly higher than those in the other groups (Figure 2).

## 4. Discussion

Given the importance of genetically modified pigs in biomedical research, an ideal environment for porcine preimplantation embryo development in vitro is required. Interdisciplinary investigations between biology and nanotechnology may solve some problems associated with biological applications, particularly IVP in pigs [2]. To provide proper culture conditions during IVP, omics technologies are being applied to discover the metabolic substrates that are activated during early embryonic processes. Therefore, combining omics and nanotechnology to develop a suitable culture medium for improving porcine IVP would increase the number of pigs that are valid for transgenic technologies. However, the use of NPs showed a considerable increase in the risk of toxic effects to the animals, because they can cross the placental barrier, leading to embryo damage [18,49].

In this experiment, we used MSmPNPs as a component to improve porcine IVM and subsequent preimplantation embryo development. Recent study showed that the biliprotein phycocyanin, derived from *Spirulina platensis*, enhanced the developmental competence of the oocytes from obese female mice [50]. Additionally, IVM supplementation with phycocyanin improved the embryonic development of parthenogenetic and cloned embryos in porcine [51]. The natural polysaccharide pectin has gained increasing attention owing to its physicochemical and biomedical activities [52]. It exerts antioxidant activity in experimental, in vitro free radical scavenging [52,53,54,55,56,57], and in vivo models [58]. Furthermore, Zhang et al. reported that lower doses (5–25 µM) of naturally occurring phenolic compounds (rosmarinic acid) attenuated intracellular ROS levels in porcine oocytes and cumulus cells during IVM [59]. Moreover, low doses (100 µM) of the polyphenolic compound procyanidin, which is derived from plant sources, also decreased ROS production and apoptosis, while promoting the quality of oocytes and PA embryo development [60]. Therefore, it is ideal to investigate the anti-oxidative effects of MSmPNPs on the critical stage of IVP in porcine IVM and subsequent embryo development.

The results showed that MSmPNPs mediate the reduction in ROS levels and increase in GSH, which are favorable conditions during IVM [61,62]. Although ROS are generated during different cellular metabolic processes, in vitro-matured oocytes are more sensitive to oxidative stress [63]. Therefore, alleviating such negative effects of oxidative stress will enhance mitochondrial function and improve oocyte maturation and cleavage capabilities [64]. Therefore, we hypothesized that pectin-like biomaterials extracted from *Spirulina maxima* may have positive anti-oxidative and free radical scavenging effects during oocyte IVM.

Previous studies have shown that SmP and SmPNPs enhance wound healing [17], disease resistance, and stress tolerance [65] by reducing oxidative stress. There is no previous report on the use of *Spirulina* pectins to improve the quality of developing oocytes. Thus, to our knowledge, this is the first study to explore the effects of marine *S. maxima* pectin on oocyte maturation and developmental competence in oocytes *in vitro*. According to our findings, the optimum concentration of MSmPNPs to effectively neutralize IVM-derived ROS in porcine oocytes was 2.5 µg/mL. Compared to the control and other treatment groups, matured oocytes in the MSmPNPs 2.5-treated group had significantly higher intracellular GSH levels and lower ROS levels. Increased intracellular GSH levels have been linked to improved cytoplasmic maturation, embryonic development, and offspring production [3]. Furthermore, GSH levels are important for maintaining the cellular redox; a lack of GSH can lead to apoptotic stimuli in mature ovarian follicles [66,67]. In the present study, 2.5 µg/mL of MSmPNPs effectively reduced intracellular ROS levels and enhanced the non-enzymatic antioxidant (GSH) level in oocytes and cumulus cells at the end of IVM.

Interestingly, IVM medium containing 2.5 µg/mL of MSmPNPs markedly improved the quality of porcine developmental competence of PA and SCNT embryos, as indicated by enhanced hatching and total cell counts of blastocysts. Moreover, qRT-PCR analysis further confirmed the upregulated expression pattern of selected reprogramming and pluripotency-related genes (*POU5F1*, *DPAA2*, and *NDP52*) at lower doses of MSmPNPs (2.5 and 5 µg/mL). From the given results, it appears that increasing the concentrations of the MSmPNPs tends to be toxic to the IVM environment and the IVM conditions favors the reduced and optimal concentration, which is 2.5 µg/mL. *PPOU5F1* (or *OCT4)* is known as a master gene for pluripotency, as its expression controls early embryonic stem cells and development [68]. *DPPA2* (or *PESCRG1)* acts as a transcription factor, is involved in the maintenance of the active epigenetic status of these genes, and maintains the pluripotency of stem cells [69,70,71]. Moreover, *NDP52* (or *CALCOCO2)* is an *OCT4*-related gene, and its expression is limited to the pluripotent cells of the early embryo and the germline (blastocysts, epiblasts, and purified primordial germ cells) and regulates embryonic stem cell pluripotency and early blastocyst development [72,73]. Collectively, the activation of *POU5F1*, *DPPA2*, and *NDP52* improved the proper reprogramming of donor nuclei after reactivation of genes [74]. Overall, the results confirmed that excess ROS neutralization by antioxidant properties of MSmPNPs could effectively improve oocyte maturation and subsequent embryonic developmental competence of cloned and parthenogenetic embryos. Further investigations are required to study the effects on in vitro fertilization-derived embryos. This finding provides novel IVM conditions through the integration of innovative nanomaterials from marine *Spirulina*.

## 5. Conclusions

Oocytes treated with MSmPNPs during IVM resulted in a higher rate of pre-implantation porcine parthenogenetic and cloned embryos development. The highest impacts were seen at a supplementation of 2.5 µg/mL MSmPNPs and had a constructive impact on oocyte quality and embryonic development and embryo eminence by increasing the levels of intracellular GSH while reducing ROS levels, as well as increasing the expression of the pluripotency-associated genes *POU5F1*, *DPPA2*, and *NDP52* in the resultant blastocysts. This finding provides novel IVM conditions through the integration of innovative nanomaterials from marine *Spirulina*.

## Figures and Tables

**Table 1:** S Specific primers used for gene expression analysis by qPCR.

Genes	Gene Full Name	Sequences (5′-3′)	Product Size (bp)	NCBI Accession No.
*ß-actin*	Beta actin	F: CCC TGG AGA GCT ACG AG	172	XM_003124280.5
		R: TCC TTC CTG ATG TCC ACG TC		
*POU5F1*	POU class 5 homeobox 1	F: AGT GAG AGG CAA CCT GGA GA	166	XM_021097869.1
		R: TCG TTG CGA ATA GTC ACT GC		
*NDP52*	Nuclear domain 10 protein	F: TGC TGA GTT ACA TGG GTC TGG	182	XM_003131552.4
		R: ACC AAG GTC TGA TTT GCA GGT		
*DPPA2*	Developmental pluripotency associated 2	F: TGA GAG AGG GGA AAA GAC CAA	151	XM_003358822.4
		R: TGG CAG AAA GGT CTC AAC AGA		

**Table 2:** Effect of in vitro maturation of porcine oocytes with different concentrations of MSmPNPs on the in vitro development rate of PA embryos.

Conc. of MSmPNPs (µg/mL)	No. of COCs	No. of Matured Oocytes (%)	No. of Embryos (Mean ± SEM)		
			Cultured	Cleaved (%)	Blastocyst (%)
0 (Control)	200	167 (83.5 ± 1.8) ^b^	161	137 (85.1 ± 1.3) ^b^	47 (29.2 ± 1.0) ^b^
2.5	200	182 (91.0 ± 1.0) ^a^	168	152 (90.5 ± 0.8) ^a^	58 (34.5 ± 1.4) ^a^
5.0	200	173 (86.5 ± 1.3) ^b^	162	140 (86.4 ± 1.3) ^a,b^	48 (29.6 ± 1.7) ^a,b^
10	200	160 (80.0 ± 0.8) ^b^	157	126 (80.3 ± 1.1) ^c^	39 (24.9 ± 1.1) ^b^

^a,b^ Values in the same column with different superscript letters are significantly different (*p <* 0.05). Number of replicates (*n* = 4).

**Table 3:** The effects of in vitro maturation of porcine oocytes with different concentrations of MSmPNPs on different cell number of in vitro PA blastocysts.

Conc. of MSmPNPs (µg/mL)	No. of Cells (Mean ± SEM)			Ratio (%) of ICM: TE
	Total Cells	TE (%)	ICM (%)	
0 (Control)	42.8 ± 1.2 ^b,c^	33.8 (78.7 ± 1.0) ^a^	9.0 (21.3 ± 1.0) ^b^	27.9 ± 1.7 ^b^
2.5	48.7 ± 1.4 ^a^	36.6 (74.8 ± 0.8) ^b^	12.0 (25.2 ± 0.8) ^a^	34.5 ± 1.4 ^a^
5.0	44.0 ± 1.1 ^b^	33.4 (75.6 ± 0.7) ^b,c^	10.7 (24.4 ± 0.7) ^a,b^	32.8 ± 1.2 ^a,b^
10	40.2 ± 1.0 ^c^	32.0 (79.6 ± 0.9) ^a^	8.2 (20.4 ± 0.9) ^b^	26.1 ± 1.5 ^b^

^a–c^ Values in the same column with different superscript letters are significantly different (*p <* 0.05). Number of replicates (*n* = 4).

**Table 4:** Effect of in vitro maturation of porcine oocytes with different concentrations of MSmPNPs on in vitro development rate of SCNT embryos.

Conc. of MSmPNPs (µg/mL)	No. of COCs	No. of Matured Oocytes (%)	No. of Embryos (Mean ± SEM)		
			Cultured	Cleaved (%)	Blastocyst (%)
0 (Control)	200	166 (83.0 ± 1.5) ^b^	137	116 (84.7 ± 1.0)	34 (24.8 ± 0.9) ^b^
2.5	200	179 (89.5 ± 1.8) ^a^	148	131 (88.5 ± 1.6)	46 (31.1 ± 1.1) ^a^
5.0	200	173 (86.5 ± 1.3) ^b^	143	125 (87.4 ± 2.1)	37 (25.9 ± 0.9) ^b^
10	200	161 (80.5 ± 1.4) ^b^	132	109 (82.6 ± 1.3)	30 (22.7 ± 1.4) ^b^

^a,b^ Values in the same column with different superscript letters are significantly different (*p <* 0.05). Number of replicates (*n* = 4).

**Table 5:** Effect of in vitro maturation of porcine oocytes with different concentrations of MSmPNPs on different cell number of SCNT blastocysts.

Conc. of MSmPNPs (µg/mL)	No. of Cells (Mean ± SEM)			Ratio (%) of ICM: TE
	Total Cells	TE (%)	ICM (%)	
0 (Control)	41.9 ± 1.9 ^b,c^	33.2 (78.7 ± 1.2) ^a^	8.8 (21.3 ± 1.2) ^b^	27.9 ± 2.2 ^b^
2.5	48.9 ± 1.5 ^a^	36.4 (74.0 ± 0.9) ^b^	12.5 (26.0 ± 0.9) ^a^	35.7 ± 1.8 ^a^
5.0	44.4 ± 1.0 ^b^	34.6 (77.7 ± 1.0) ^a^	9.7 (22.3 ± 1.0) ^b^	29.0 ± 1.7 ^b^
10	39.2 ± 2.5 ^c^	31.0 (78.5 ± 1.1) ^a^	8.2 (21.5 ± 1.1) ^b^	27.6 ± 1.7 ^b^

^a–c^ Values in the same column with different superscript letters are significantly different (*p <* 0.05). Number of replicates (*n* = 4).
